# NEMA NU 1-2018 performance characterization and Monte Carlo model validation of the Cubresa Spark SiPM-based preclinical SPECT scanner

**DOI:** 10.1186/s40658-023-00555-6

**Published:** 2023-06-01

**Authors:** Matthew E. Strugari, Drew R. DeBay, Steven D. Beyea, Kimberly D. Brewer

**Affiliations:** 1 Biomedical Translational Imaging Centre, Halifax, NS Canada; 2 Department of Physics and Atmospheric Science, Dalhousie University, Halifax, NS Canada; 3 Cubresa Inc., Winnipeg, MB Canada; 4 Department of Diagnostic Radiology, Dalhousie University, Halifax, NS Canada; 5 School of Biomedical Engineering, Dalhousie University, Halifax, NS Canada; 6 Department of Microbiology and Immunology, Dalhousie University, Halifax, NS Canada

**Keywords:** Molecular imaging, Nuclear medicine, SPECT, Animal imaging instrumentation, Monte Carlo method, Computer-assisted image processing, Imaging phantoms

## Abstract

**Background:**

The Cubresa Spark is a novel benchtop silicon-photomultiplier (SiPM)-based preclinical SPECT system. SiPMs in SPECT significantly improve resolution and reduce detector size compared to preclinical cameras with photomultiplier tubes requiring highly magnifying collimators. The NEMA NU 1 Standard for Performance Measurements of Gamma Cameras provides methods that can be readily applied or extended to characterize preclinical cameras with minor modifications. The primary objective of this study is to characterize the Spark according to the NEMA NU 1-2018 standard to gain insight into its nuclear medicine imaging capabilities. The secondary objective is to validate a GATE Monte Carlo simulation model of the Spark for use in preclinical SPECT studies.

**Methods:**

NEMA NU 1-2018 guidelines were applied to characterize the Spark’s intrinsic, system, and tomographic performance with single- and multi-pinhole collimators. Phantoms were fabricated according to NEMA specifications with deviations involving high-resolution modifications. GATE was utilized to model the detector head with the single-pinhole collimator, and NEMA measurements were employed to tune and validate the model. Single-pinhole and multi-pinhole SPECT data were reconstructed with the Software for Tomographic Image Reconstruction and HiSPECT, respectively.

**Results:**

The limiting intrinsic resolution was measured as 0.85 mm owing to a high-resolution SiPM array combined with a 3 mm-thick scintillation crystal. The average limiting tomographic resolution was 1.37 mm and 1.19 mm for the single- and multi-pinhole collimators, respectively, which have magnification factors near unity at the center of rotation. The maximum observed count rate was 15,400 cps, and planar sensitivities of 34 cps/MBq and 150 cps/MBq were measured at the center of rotation for the single- and multi-pinhole collimators, respectively. All simulated tests agreed well with measurement, where the most considerable deviations were below 7%.

**Conclusions:**

NEMA NU 1-2018 standards determined that a SiPM detector mitigates the need for highly magnifying pinhole collimators while preserving detailed information in projection images. Measured and simulated NEMA results were highly comparable with differences on the order of a few percent, confirming simulation accuracy and validating the GATE model. Of the collimators initially provided with the Spark, the multi-pinhole collimator offers high resolution and sensitivity for organ-specific imaging of small animals, and the single-pinhole collimator enables high-resolution whole-body imaging of small animals.

## Introduction

Functional imaging in nuclear medicine extensively employs positron emission tomography (PET) and single-photon emission computed tomography (SPECT) for disease diagnosis and staging, therapy planning, dosimetry, and monitoring of treatment response [1, 2]. These nuclear medicine techniques are based on radiopharmaceutical uptake within the body, yielding critical diagnostic information that can readily translate to developing theranostic strategies for managing various diseases [3, 4]. Such investigations are commonly performed in the preclinical setting to evaluate the effects of novel drugs and therapies in small animals, which requires that small animals be the appropriate surrogate for humans [5]. Mice are one of the preferred species for biomedical research because of their anatomical, physiological, and genetic similarity to humans [6]. Furthermore, preclinical imaging with mice demands high-resolution technology due to the study of relatively small organs that are approximately 3,000 times smaller in mice than humans [7]. Recent advancements in imaging technology have promoted widespread adoption of small-animal imaging, and the availability of dedicated preclinical scanners has increased to satisfy this demand. Some advantages of SPECT over PET include superior spatial resolution, simultaneous multi-energy and multi-isotope signature detection, increased accessibility to isotopes with a range of physical half-lives, relatively simple and stable radiochemistry with increased specific activities, and reduced production costs [8, 9]. Therefore, developing sensitive and accurate preclinical SPECT systems is of growing importance.

Monte Carlo simulations are also essential in emission tomography investigations to model, develop, and evaluate nuclear-based imaging systems [10]. The Monte Carlo method is considered the gold standard for designing new medical imaging devices, offering an effective means to assess performance, optimize acquisition protocols, and evaluate new image reconstruction algorithms and correction techniques. Several Monte Carlo packages exist including Geometry and Tracking (Geant4) [11], Electron Gamma Shower (EGS) [12], and Monte Carlo N-Particle (MCNP) [13], all of which provide well-validated physics models and geometry modelling tools. These toolkits focus on radiation transport simulations, and tuning the code to model PET and SPECT devices can be challenging. The Geant4 Application for Tomographic Emission (GATE) aims to simplify the modelling process while accommodating complex scanner geometries and imaging configurations using geometric definitions, time-dependent phenomena, radioactive source definitions, detector electronics modelling, and data output [10].

Several commercially available preclinical SPECT detectors have been investigated with GATE, including X-SPECT (TriFoil Imaging, Chatsworth, USA) [14], Inveon (Siemens, Munich, Germany) [15], HiReSPECT (Parto Negar Persia, Tehran, Iran) [16], and NanoSPECT/CT^PLUS^ (Mediso, Budapest, Hungary) [17] scanners. These systems, like all SPECT systems, are constructed with varying component designs, including but not limited to pinhole or parallel-hole collimators, monolithic or pixelated scintillation crystals, and solid-state or vacuum tube detector technologies. Cameras that use photomultiplier tubes (PMTs) for high-resolution preclinical SPECT are large and bulky and require highly magnifying pinhole collimators to overcome the limiting intrinsic spatial resolution of PMTs. While position-sensitive PMTs (PSPMTs) offer a smaller form factor than PMTs with improved resolution, their combination with scintillation crystals to detect $\gamma$- and X-rays yields a detector that is also several centimeters thick, and the camera size is further increased when attaching pinhole or parallel-hole collimators. Recent advancements in solid-state technology, such as cadmium zinc telluride (CZT) for direct detection or silicon-photomultipliers (SiPMs) coupled with scintillation crystals for indirect detection, provide advantages over PMT-based technology, including a smaller form factor for design flexibility, superior intrinsic spatial resolution, reduced power consumption, and insensitivity to magnetic fields and vibrations [18]. The use of SiPMs in SPECT is becoming more established as demonstrated in the literature by a large area clinical SPECT detector [19] and SPECT inserts for clinical and preclinical magnetic resonance imaging [20–22]. An example of a novel SiPM-based preclinical SPECT scanner is the Cubresa Spark (Cubresa Inc., Winnipeg, Canada) [23, 24].

Cubresa’s implementation of SiPMs in a pinhole-SPECT system, with a magnification factor near unity and a form factor small enough for benchtop use, is a novel application of SiPMs in SPECT evaluated in the current study. The Spark is a small-animal benchtop SPECT system optimized for *in vivo* mouse imaging and can be configured with up to two detector heads. Its current configuration features one detector head, single- and multi-pinhole collimators, a sodium-activated cesium iodide (CsI(Na)) scintillation crystal, and a SiPM array to achieve high-resolution planar and tomographic imaging. Altogether, the detector head is less than 6 cm-thick from the face of the collimator to the exterior of the back compartment housing the electronics. This allows the Spark to be attached to preclinical computed tomography (CT) scanners for multi-modal disease study, translational research, and drug discovery applications. For example, the Spark was recently utilized in developing diagnostic radiopharmaceuticals for Alzheimer’s disease [25]. Due to the limited yet increasing use of SiPMs in SPECT, the performance characteristics of a preclinical SiPM SPECT scanner have not been established or compared to other scanners in the literature.

To compare different $\gamma$-cameras, the National Electrical Manufacturers Association (NEMA) has published the NEMA NU 1-2018 Standard for Performance Measurements of Gamma Cameras [26]. This standard provides a uniform and consistent method for measuring and reporting performance parameters for various camera designs. Although NEMA has published a *clinical* and *preclinical* standard for PET scanners, a preclinical SPECT standard is currently unavailable. However, the NEMA NU 1-2018 clinical standard can be readily applied or extended to preclinical SPECT cameras with minor modifications. NEMA standards also provide a rigorous and thorough approach to validating Monte Carlo models, unlike some previously modelled systems in GATE that used widely varying, incomplete, or untraceable validation approaches.

The primary objective of this study is to evaluate the performance characteristics of a high-resolution SiPM-based preclinical SPECT scanner—the Cubresa Spark—according to the NEMA NU 1 Standard for Performance Measurements of Gamma Cameras. A secondary objective is to configure and validate a GATE simulation model of the Spark using the measured NEMA results. Collectively, this study aims to accurately establish the imaging performance of a SiPM SPECT system in planar and tomographic acquisitions offering critical insight into its utility in supporting the development of novel molecular imaging agents and techniques.

## Methods and materials

### Equipment description

**Fig. 1:**
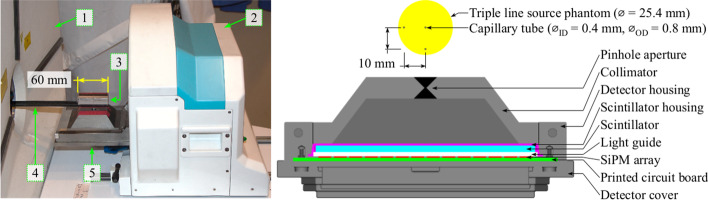
The Cubresa Spark preclinical SPECT scanner and mouse-sized NEMA triple line source scatter phantom illustrated in a photograph of the system (left) and an axial cutaway view of the detector head modelled in GATE (right). The labelled components in the photograph are the Triumph LabPET4/CT (1), Cubresa Spark gantry (2), mouse-sized NEMA triple line source scatter phantom (3), the animal bed (4), and the SPECT detector head (5). The triple line source phantom dimensions are included for scale

The Spark (Fig. 1) was affixed to the benchtop of a Triumph LabPET4/CT dual-modality system (TriFoil Imaging, Chatsworth, USA), and although the Triumph’s imaging systems were unused in this study, the animal bed was used for positioning radioactive source distributions in SPECT tests. The Spark’s detector housing, detector cover, and collimator were manufactured from tungsten that yield an overall length, width, and height of $150.4\times 138.1\times 56.4\,\hbox {mm}^{3}$ when assembled. The detector housing accepts an aluminum scintillator housing assembled with a $102\times 102\times 3\,\hbox {mm}^{3}$ monolithic CsI(Na) scintillation crystal (Saint-Gobain Crystals, Hiram, USA) and a 2 mm-thick glass light guide. Saint-Gobain BC-631 silicone grease was used to optically couple the light guide to a $14\times 14$ SensL C-series SiPM array comprised of 6 mm sensors with a 7.2 mm pitch on a printed circuit board (ON Semiconductor, Phoenix, USA). The SiPM array operates at room temperature without a cooling system. Further information regarding the construction of the Spark may be obtained from the manufacturer.

As outlined in Table 1, the Spark performance was assessed with two interchangeable tungsten collimators (Scivis GmbH, Göttingen, Germany): a single-pinhole (SPH) collimator for high-resolution planar and tomographic imaging, and a multiplexing multi-pinhole (MPH) collimator for high-resolution tomography with increased sensitivity. The SPH collimator has a non-focusing right-circular double-cone pinhole, and the MPH collimator uses a $5\times 5$ array of focusing right-circular double-cone pinholes where each row focuses on a different volume of interest (VOI) in the tomographic field of view (FOV) [27]. The area of the detector used for imaging $\gamma$- and X-rays has a useful field of view (UFOV) and central field of view (CFOV) of 84.5 mm and 63.375 mm, respectively.

**Table 1:** Geometric specifications of pinhole collimators

Aperture^a^	SPH	MPH
Pinhole diameter (mm)	1.0	1.0
Pinhole acceptance angle ($^{\circ }$)	90.0	25.0
Number of pinholes	1	25
Thickness (mm)	10.0	10.0
Radius of rotation (mm)	28.0	28.0
Aperture-to-detector distance^b^ (mm)	26.75	26.75
Reconstructed axial FOV (mm)	57.0	14.0
Reconstructed transaxial FOV (mm)	46.0	30.0

^a^ SPH: single-pinhole, MPH: multi-pinhole

^b^ Measured to face of scintillation crystal

The Spark was delivered with Scivis’ HiSPECT reconstruction software, which was preconfigured solely for the MPH collimator. Precise information regarding the MPH collimator geometry was not readily available, and as a result, this restricted the simulation model to the SPH collimator only. Measured and simulated SPH SPECT images were reconstructed with the Software for Tomographic Image Reconstruction (STIR) v5.1.0 using the pinhole-SPECT acquisition matrix [28–30].

Prior to measurement, the SPECT system was calibrated for energy, linearity, uniformity, center of rotation, and aperture-to-detector distance [31, 32]. Radionuclide activity measurements were performed with a Capintec CRC-55tR dose calibrator (Mirion Technologies, Florham Park, USA). Various phantoms and source positioning jigs were fabricated in-house to adhere to the NEMA protocol, and each required device is described in the following sections.

### Simulation description

A model of the Spark detector head (Fig. 1) was created using the SPECThead system in the GATE v9.0 Monte Carlo toolkit [10] compiled with Geant4 10.06.p01 [11] and Rapid Object-Oriented Technology (ROOT) 6.14.04 [33]. Simulations were distributed over 12 cores on an HP Z820 workstation operating Ubuntu 18.04.5 LTS with two Intel Xeon E5-2630 2.3 GHz hexa-core CPUs and 64 GB of 1600 MHz DDR3 memory. ROOT output was combined into one file and then converted to Cubresa’s list mode format for further processing.

Complex detector geometry was modelled with standard tessellation language (STL) files provided by Cubresa, and simple geometric volumes such as the scintillator, light guide, SiPM array, printed circuit board, and phantoms were modelled with predefined shapes available in GATE. Material properties were assigned to their respective volumes using the Geant4 and GATE materials database. More specifically, the modelled collimator, detector housing, and detector cover materials were tungsten, the scintillator housing was aluminum, the scintillation crystal was CsI, the light guide was glass, the SiPM array was silicon, and the printed circuit board was epoxy. The scintillation process, optical photon transport, and light detection were not simulated to save computing time. Therefore, the silicone optical grease was negated from the simulation model. Other excluded components were the 3.5 mm-thick carbon fiber animal bed due to its application in only two NEMA tests with minimal attenuation in SPECT acquisitions, and the MPH collimator due to restricted knowledge of the pinhole geometry. For reasons detailed in the Discussion, the SPH collimator was modelled with a 0.85 mm-diameter pinhole to better match the simulated collimator-detector response function to measurement.

**Fig. 2:**
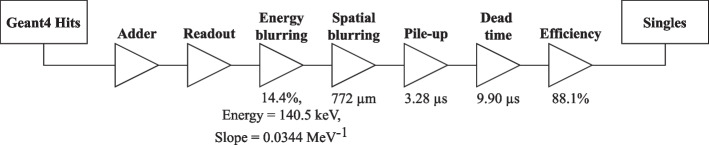
Digitizer signal processing model of the Spark’s readout electronics used in GATE. Interactions in the scintillation crystal were recorded as hits following Geant4 particle generation and transport through modelled materials. Hits were subsequently filtered through the digitizer modules to obtain singles corresponding to the detected signal after processing by the front-end electronics. Digitizer parameters were determined empirically from measurement by simulating a range of values for a given digitizer parameter, fitting a cubic spline to the simulated results, then interpolating the digitizer parameter at the measured result, except for the pile-up timing resolution which was calculated with Eq. 1

Physics processes were initialized with the Geant4 standard electromagnetic physics package option 4 (emstandard_opt4) [11]. Particle production cuts were set at the default value of 1 mm corresponding to a few keV in most materials, except the scintillation crystal and pinhole knife-edge where the threshold was set to 1 keV. Radioactive sources were defined as an isotropic UserSpectrum source of $\gamma$-rays with emissions defined from the Table of Radionuclides [34]. The Spark’s electronics, i.e., signal processing chain, were modelled using the following GATE digitizer modules: the adder, readout, energy blurring, spatial blurring, pile-up, dead time, and efficiency. Figure 2 presents the digitizer chain with the values set for parameters of interest. Digitizer parameters were determined empirically from measurement by simulating a range of values for a given digitizer parameter, fitting a cubic spline to the simulated results, then interpolating the digitizer parameter at the measured result. However, the pile-up timing resolution $t_\text {min}$ was calculated as

1 $$\begin{aligned} t_\text {min} = \frac{P_1}{R_\text {T} (P_0 + 2P_1)} \end{aligned}$$

where $P_0$ and $P_1$ are the counts in the primary and first order pile-up peaks, respectively, and $R_\text {T}$ is the true input count rate [35].

### NEMA performance characterization and SPECT model validation

Performance characterization of the Spark was made according to the NEMA NU 1-2018 protocol, with tests briefly described in the following sections. The radionuclide for all tests was technetium-99m (${}^\text {99m}$Tc) except for the Multiple Window Spatial Registration test which used Gallium-67 ($^{67}$Ga). An energy window width of 30% was centered on the reference photopeak(s) when generating projection images for all tests. The UFOV and CFOV were defined with electronic masking, and images had 0.1 mm isotropic pixels unless stated otherwise. Measured data were acquired according to total acquisition time or counts through an open energy window. Note that acquired counts refer to the computer’s unprocessed estimate of counts determined from the optical light collected from the scintillation crystal, which Cubresa’s proprietary data processing software then converts to detected/observed counts stored in list mode data in terms of position, energy, and time. Simulations were then configured based on measurements of data acquisition time, radioactivity, radioactive source distribution, and system geometry, except for the SPH collimator pinhole diameter. No corrections were applied to the simulated data at any stage. Validation of the GATE model was based on reporting parameter comparisons between measured and simulated NEMA results.

### Tests of intrinsic gamma camera detector characteristics

#### Intrinsic spatial resolution and linearity

Intrinsic spatial resolution refers to the $\gamma$-camera’s ability to localize an ionizing photon’s interaction site within the detector, and intrinsic linearity reflects the distortion of those interaction sites throughout the detector’s FOV. This test was performed with a 2.5 mm-thick tungsten planar mask comprised of a $3\times 3$ grid of 0.8 mm-wide and 26.5 mm-long parallel slits having adjacent slit centers separated by 31.5 mm, and a Derenzo pattern with {0.7, 0.8, 0.9, 1.0, 1.2, 1.4} mm-diameter holes. An Eppendorf tube containing a 50 MBq point source was centered 65 cm above the face of the detector, and 15 million counts were acquired. Intrinsic resolution and linearity were assessed from line spread functions (LSFs) and analyzed according to the procedures defined by the NEMA NU 1-2018 protocol. A millimeters-per-pixel calibration factor was also calculated using line profile spacing to convert relevant image dimensions to physical units in relevant NEMA tests.

Normally, the mask-slit geometry would yield the limiting intrinsic spatial resolution. However, due to the spatial resolution performance of the SiPM detector, the mask-slit geometry described above produced LSFs that were wider than the intrinsic spatial resolution. Therefore, a secondary test was performed using a non-NEMA source geometry to extract the limiting resolution. A point spread function (PSF) was created with a pencil beam emitted from a tungsten line source holder with a tunnel 0.4 mm in diameter, 10.0 mm in length, and centered 1.0 mm above the middle of the detector with a 1.0 cm-thick aluminum plate. A total of 100,000 counts were acquired from a 170 MBq line source established in a glass capillary tube (inner diameter $\varnothing _\text {ID}=1.15\,\hbox {mm}$, outer diameter $\varnothing _\text {OD}=1.50\,\hbox {mm}$, length $L=75\,\hbox {mm}$) and secured in the line source holder. The PSF was then analyzed following the methods applied to the LSFs produced with the mask-slit geometry.

#### Intrinsic flood field uniformity

The intrinsic uniformity quantifies the $\gamma$-camera’s response to a uniform radiation flux. An 8 MBq point source was centered 65 cm above the face of the detector, and 100 million counts were acquired. The measured and simulated flood field projection images with 1 mm pixels were smoothed once by convolution with the NEMA smoothing filter, and measured data were corrected for uniformity. The integral uniformity was calculated using

2 $$\begin{aligned} \text {Uniformity}\,(\%) = \frac{\text {max} - \text {min}}{\text {max} + \text {min}} \times 100 \end{aligned}$$

where $\text {max}$ and $\text {min}$ refer to the maximum and minimum pixel values within the FOV. Similarly, the differential uniformity was calculated with Eq. 2 from the $\text {max}$ and $\text {min}$ in a set of five contiguous pixels in a row or column.

#### Multiple window spatial registration

The multiple window spatial registration (MWSR) test was performed with 11 MBq of ${}^\text {67}$Ga to assess the Spark’s ability to accurately localize photons of different energies when imaged through different energy windows. The previously described pencil beam source holder (see Intrinsic spatial resolution and linearity) was positioned in a 1.0 cm-thick aluminum plate at nine locations along the detector axes, including the middle of the detector, $0.4\times$, and $0.8\times$ the distance to the edge of the UFOV. A total of 4 million counts were acquired at each position, and projection images were generated from each photopeak. The maximum axial and transaxial displacements of PSF centroids were then calculated. Overall spatial registration accuracy was also assessed according to the mean Euclidean distance between each centroid and the average centroid location.

#### Intrinsic count rate performance in air: decaying source method

The count rate performance describes the $\gamma$-camera’s ability to process one detection event before moving on to another, and the number of detected counts may be fewer than input events because of dead time and/or pile-up. Two models exist to describe idealized dead time behaviour: paralyzable and non-paralyzable dead time [36]. The Spark’s behaviour is well described with a paralyzable model using the equation

3 $$\begin{aligned} \text {OCR} = \text {ICR}\,\textrm{e}^{-\text {OCR}\tau } \end{aligned}$$

where $\text {OCR}$ is the observed count rate, $\text {ICR}$ is the input count rate, and $\tau$ is the system dead time. Furthermore, $\text {OCR}$ can be affected by pile-up, which occurs when a true event at time $t=0$ is followed by subsequent events in the interval $0<t<\tau$, followed by an event-free interval of length $\tau$. Using the decaying source method, the dead time was calculated from the intercept and slope of Eq. 4:

4 $$\begin{aligned} \lambda t + \ln \text {OCR} = -\text {ICR}_0 \tau \textrm{e}^{-\lambda t} + \ln \text {ICR}_{0} \end{aligned}$$

where $\lambda$ is the decay constant, *t* is the time, $\text {ICR}_0$ is the true input rate at the beginning of measurement, $e^{-\lambda t}$ is the abscissa, and $\lambda t + \ln \text {OCR}$ is the ordinate [36].

Care was taken to minimize scatter during count rate performance assessment by securing an Eppendorf tube containing 235 MBq in a tungsten Capintec 511 Dose Drawing Syringe Shield. The shield was capped with a lead lid, and a 6.0 mm-thick copper plate covered the open side of the source holder. The source was placed at a distance of $5\times$ UFOV above the detector face to produce a uniform radiation field. Counts were measured for 60 s and simulated for 10 s in 60 min intervals, and the last data point was acquired when the observed count rate dropped below 600 cps to determine $\text {ICR}_0$ accurately. All data were corrected for radioactive decay, and the measured data were corrected for background noise and uniformity. Measured count rate data were utilized to configure the digitizer pile-up, dead time, and efficiency modules in the simulation model. Following the NEMA protocol, the intrinsic count rate performance was analyzed in terms of the maximum $\text {OCR}$ and 20% loss $\text {OCR}$.

#### Intrinsic energy resolution

The energy resolution characterizes a radiation detector’s response to a monoenergetic radiation source and describes its ability to distinguish between different energies of that radiation. The formal definition is

5 $$\begin{aligned} \text {Energy resolution}\, (\%) = \frac{\text {FWHM}}{\text {Photopeak location}} \times 100 \end{aligned}$$

where FWHM is the full width at half maximum of the photopeak calculated according to NEMA’s resolution methodology in this context. The Spark’s intrinsic energy resolution was assessed using 0.6 keV bins with the count rate data point immediately below the 20% loss OCR introduced in the previous section (Intrinsic count rate performance in air: decaying source method). This data point satisfies all NEMA conditions while offering count rate traceability. The simulated data point below the 20% loss OCR was re-simulated with a 60 s acquisition time to obtain count statistics comparable to the measurement. Note that a keV-per-channel calibration factor was not calculated with cobalt-57 (${}^\text {57}$Co) since a vendor-specific energy calibration is automatically applied to list mode data.

### Tests of gamma camera detectors with collimators

In this study, system or *extrinsic* measurements primarily involved the SPH collimator due to its applicability in planar scintigraphy yielding unambiguous projection images. Measurements with the experimental MPH were included where applicable.

#### System spatial resolution without scatter

The system spatial resolution without scatter represents the $\gamma$-camera’s limiting ability to localize a photon interaction site in the detector when combining collimator and intrinsic factors. Acquisitions were performed in the axial and transaxial directions using a precision glass capillary tube ($\varnothing _\text {ID}=0.4\,\hbox {mm}$, $\varnothing _\text {OD}=0.8\,\hbox {mm}$, $L=75\,\hbox {mm}$). The capillary tube contained 10 MBq of radioactivity, and 100,000 counts were acquired at positions of {0.4, 25.0, 50.0, 75.0, 100.0} mm from the face of the SPH collimator. NEMA’s resolution methodology was applied to calculate resolution from LSFs. Results were corrected for magnification to compare resolution in the object rather than the detector. A plot of the average system resolution as a function of source-to-collimator distance was generated with a linear least squares fit to characterize the system resolution.

#### System spatial resolution with scatter

The presence of a scattering medium degrades image quality in terms of projection image blurring, reduced contrast in reconstructed images, and decreased quantitative accuracy [37]. Thus, the system spatial resolution with scatter was assessed with a mouse-sized NEMA triple line source scatter phantom fabricated from an acrylic cylinder ($\varnothing =25.4\,\hbox {mm}, L=60\,\hbox {mm}$) with three 0.8 mm-diameter bores for precision capillary tubes: one at the center and two separated by 90$^{\circ }$ with a 10 mm radial offset. One precision capillary tube containing 10 MBq was inserted into the central bore of the scatter phantom, and 100,000 counts were acquired axially and transaxially at capillary tube positions of {12.7, 25.0, 50.0, 75.0, 100.0} mm from the face of the collimator. Analysis of the resulting projection images followed the methods outlined in System spatial resolution without scatter.

#### System planar sensitivity

The system planar sensitivity characterizes the number of detected counts per unit activity to evaluate a collimator’s count rate performance. A 35.0 mm-diameter petri dish was filled with a solution of 2 ml of water and injected with a calibrated activity of $A_\text {cal}=210\,\text {MBq}$ for the SPH dataset and $A_\text {cal}=25\,\text {MBq}$ for the MPH dataset. The internal base of the radioactive solution was placed at source-to-collimator distances of $D=\{10.0, 20.0, 28.0, 50.0, 100.0\}\,\hbox {mm}$, and 4 million counts were acquired at each position in measurement. In contrast, counts were acquired for 100 s at each position in simulation to save on computing time. Data were acquired from the largest to the smallest distance with activity levels ranging from $A_\text {cal}$ to $\sim$15 MBq to minimize pile-up and dead time effects, namely in the SPH acquisition. Measured data were corrected for uniformity, and then, the decay-corrected count rate *R* was calculated for each acquisition *i* as

6 $$\begin{aligned} R_i = \lambda C_i \textrm{e}^{\lambda (T_i - T_\text {cal})} \times \big (1-\textrm{e}^{-\lambda T_{\text {acq},i}}\big )^{-1} \end{aligned}$$

where $C_i$ is the summed counts from the projection image, $T_i$ is the acquisition start time, $T_{\text {acq},i}$ is the acquisition duration, and $T_\text {cal}$ is the time of activity calibration. Using a standard Levenberg–Marquardt nonlinear least squares fit technique, the decay-corrected count rate and source-to-collimator distance for each SPH acquisition were fit with the function

7 $$\begin{aligned} R_i = c_0 + c_1 \textrm{e}^{(-c_2D_i)} \end{aligned}$$

where $c_0$, $c_1$, and $c_2$ are fitting parameters. The total system sensitivity $S_\text {TOT}$ was then calculated as

8 $$\begin{aligned} S_{\text {TOT},i} = \frac{R_i}{A_\text {cal}} \end{aligned}$$

and plotted against the source-to-collimator distance to characterize the sensitivity. Note that NEMA’s protocol utilizes fit parameters from Eq. 7 to compute a collimator penetration factor for detected counts in a given region of interest (ROI). This analysis was excluded as it does not apply to pinhole collimators. Furthermore, Eq. 7 does not apply to the MPH collimator due to the focusing orientation of pinholes.

### Tests specific to tomographic camera systems

SPECT projection data were acquired from $0^{\circ }$ to $270^{\circ }$ in a $208\times 208$ matrix with 0.5 mm isotropic pixels and then reconstructed with nine iterations of the maximum likelihood expectation maximization (MLEM) algorithm in 0.25 mm isotropic voxels. SPH SPECT data were acquired in $3^{\circ }$ increments and then reconstructed with STIR in a $230\times 184\times 184$ matrix, and MPH SPECT data were acquired in $90^{\circ }$ increments and then reconstructed with HiSPECT in an $80\times 144\times 144$ matrix. HiSPECT software only supports the MLEM algorithm, whereas STIR’s pinhole-SPECT software permits access to STIR’s extensive library of algorithms and corrections for the spatially variant collimator-detector response and attenuation. Thus, SPH SPECT data were also reconstructed with the filtered back projection (FBP) algorithm using a ramp filter to adhere to the NEMA protocol.

#### SPECT reconstructed spatial resolution without scatter

The reconstructed spatial resolution without scatter reflects the limiting size of a radioactive distribution that can be observed with the $\gamma$-camera. Three point sources in air were established in precision capillary tubes with a mean activity of $0.274\pm 0.007$ MBq and an axial extent of $\sim$0.4 mm. To conform to the small reconstructed FOV of the MPH collimator (see Table 1), one point source was centered on the axis of rotation, and the two remaining point sources were positioned at ± 75% of the distance to the edge of the FOV, i.e., ± 5.25 mm axially and ± 11.25 mm transaxially. The point sources were set in place, and 300,000 counts were acquired across all projections in the SPH and MPH acquisitions to directly compare tomographic resolution. Cubic ROIs were centered around each reconstructed point source and summed along each axis to calculate the radial, tangential, and axial resolution without scatter according to the NEMA protocol.

#### SPECT reconstructed spatial resolution with scatter

The reconstructed spatial resolution with scatter was assessed with the mouse-sized NEMA triple line source scatter phantom described in System spatial resolution with scatter. Three capillary tubes containing a mean activity of $9.4\pm 0.1$ MBq were inserted into the phantom and centered axially in the FOV with peripheral line sources placed at $0^{\circ }$ and $270^{\circ }$ to maximize the amount of scatter contributing to projection images over the extent of rotation. The line sources in the scatter phantom were set in place, and 5 million counts were acquired across all projections in the SPH and MPH acquisitions to directly compare tomographic resolution. The reconstructed images were summed axially to obtain three 3.5 mm-thick transverse slices: one at the center of the FOV and two at ± 75% the distance to the edge of the respective axial FOV. A square ROI was centered on each resulting PSF to calculate the central, radial, and tangential resolution with scatter according to the NEMA protocol.

#### SPECT volume sensitivity, uniformity, and variability

The system volume sensitivity (SVS) reports the total system sensitivity to a uniform activity concentration in a cylindrical phantom. An acrylic phantom ($\varnothing _\text {ID}=26\,\hbox {mm}$, $\varnothing _{\text {OD}}=28\,\hbox {mm}$, $L_\text {inner}=21\,\hbox {mm}$) was filled with water containing 1.75 MBq/ml then centered along the axis of rotation in the $\gamma$-camera’s image space. The phantom was set in place, and SPH and MPH SPECT acquisitions were obtained with 10 s and 60 s projections, respectively. The measured data were corrected for uniformity, then the SVS was calculated as

9 $$\begin{aligned} \text {SVS} = \frac{A}{B_\text {c}} \end{aligned}$$

where *A* is the average count rate (total detected counts divided by total elapsed time including time for rotation) and $B_\text {c}$ is the activity concentration halfway through the acquisition. By normalizing the $\text {SVS}$ by the axial extent *L* of the cylindrical phantom in the reconstructed image, the volume sensitivity per axial centimeter ($\text {VSAC}$) was calculated as

10 $$\begin{aligned} \text {VSAC} = \frac{\text {SVS}}{L}. \end{aligned}$$

The $\text {VSAC}$ was then multiplied by the reconstructed axial FOV of the collimator to obtain a useful approximation of the total system response to a broad distribution of radioactivity.

Although it is not a defined NEMA test, the volume uniformity was evaluated from images of the cylindrical phantom reconstructed with the MLEM algorithm. Integral uniformity was calculated with Eq. 2 from a VOI covering 75% of the phantom’s imaged length and 60% of the phantom’s inner diameter. Within this VOI, the variability was determined from the coefficient of variation (CV):

11 $$\begin{aligned} \text {CV}\,(\%) = \frac{\sigma }{\mu } \times 100 \end{aligned}$$

where $\sigma$ is the standard deviation and $\mu$ is the mean voxel value within the VOI.

## Results

### Tests of intrinsic gamma camera detector characteristics

#### Intrinsic spatial resolution and linearity

**Fig. 3:**
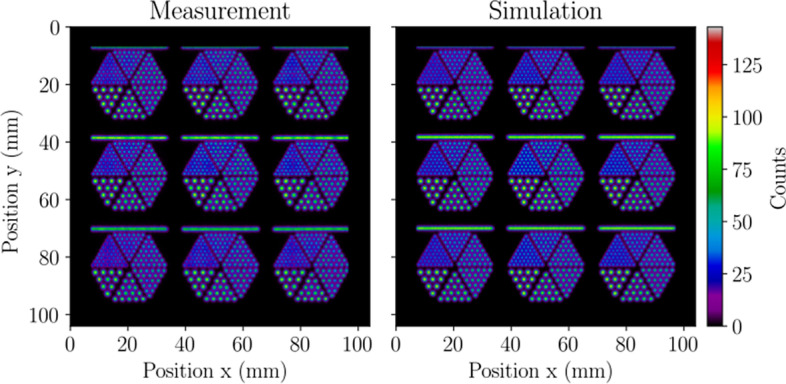
Representative planar mask projection images from measurement (left) and simulation (right) with 0.1 mm isotropic pixels and without uniformity correction. The measured image agrees well with the simulation and demonstrates the minimal distortion and superior resolution of SiPMs. The Derenzo patterns are fully resolved, and the FWHM of PSFs produced by the 0.7 mm-diameter holes was consistent with a limiting intrinsic resolution of 0.85 mm. The images shown are for demonstrative purposes as the detector’s FOV is not large enough to include the full extent of all line profiles and Derenzo patterns, which resulted in clipping of the line profiles shown at $y=7$ mm. Therefore, when analyzing the intrinsic spatial resolution and linearity from all line profiles, the central line profiles were placed across the center of the detector as instructed by the NEMA protocol, thereby clipping the Derenzo pattern at $y=97$ mm (not shown)

Representative planar mask projection images from measured and simulated acquisitions are presented in Fig. 3, and Table 2 gives the intrinsic spatial resolution determined from the pencil beam PSF and planar mask LSFs in terms of the FWHM and full width at tenth maximum (FWTM). The pencil beam produced a measured and simulated limiting intrinsic spatial resolution of 0.85 mm, which was $\sim$ 7% below that predicted by the planar mask slits. Table 2 also presents the differential and absolute intrinsic spatial linearity results, which were found to be $\lesssim$ 0.1 mm in measurement and simulation. The measured and simulated linearity results calculated a calibration factor of 0.099 mm/pixel. Altogether, good agreement was observed between measurement and simulation, and measured results indicated highly accurate positioning and minimal distortion of detected photons with the SiPM array.

**Table 2:** Intrinsic spatial resolution and linearity

Reporting parameter	Region of interest	Measurement	Simulation
Resolution PSF FWHM (mm)	Middle of FOV	$0.851\pm 0.010$	$0.850\pm 0.003$
Resolution PSF FWTM (mm)	Middle of FOV	$1.559\pm 0.014$	$1.591\pm 0.007$
Resolution LSF FWHM (mm)	UFOV	$0.912\pm 0.098$	$0.916\pm 0.026$
	CFOV	$0.953\pm 0.091$	$0.924\pm 0.029$
Resolution LSF FWTM (mm)	UFOV	$1.73\pm 0.15$	$1.66\pm 0.03$
	CFOV	$1.80\pm 0.14$	$1.68\pm 0.03$
Differential linearity (mm)	UFOV	0.023	0.001
	CFOV	0.024	0.002
Absolute linearity (mm)	UFOV	0.102	0.003
	CFOV	0.055	0.003

#### Intrinsic flood field uniformity

Integral and differential uniformities calculated from the UFOV and CFOV of flood field images are presented in Table 3. The measured and simulated uniformity results were < 3% and < 2%, respectively, showing good agreement and uniform response to radiation.

**Table 3:** Flood field uniformity

Reporting parameter	Region of interest	Measurement	Simulation
Integral uniformity (%)	UFOV	2.96	1.72
	CFOV	2.79	1.96
Row differential uniformity (%)	UFOV	2.75	1.69
	CFOV	2.52	1.77
Column differential uniformity (%)	UFOV	2.75	1.67
	CFOV	2.11	1.67

#### Multiple window spatial registration

The higher energy $\gamma$-rays from $^{67}$Ga were observed to penetrate the walls of the tungsten pencil beam holder and produce noisy projection images, resulting in a significant fraction of total counts detected outside the pencil beam PSF. Nonetheless, the measured (simulated) MWSR was found to have maximum PSF centroid displacements in the axial and transaxial directions of 0.192 mm (0.095 mm) and 0.259 mm (0.149 mm), respectively, which reflects the worst-case scenarios of misregistration. The mean Euclidean distance between each centroid and the average centroid location for a given pencil beam location was $0.050\pm 0.023$ mm and $0.044\pm 0.022$ mm in measurement and simulation, respectively. In other words, photons of different energies were accurately localized, and centroids from different energy windows were found within one pixel of each other on average.

#### Intrinsic count rate performance in air: decaying source method

**Fig. 4:**
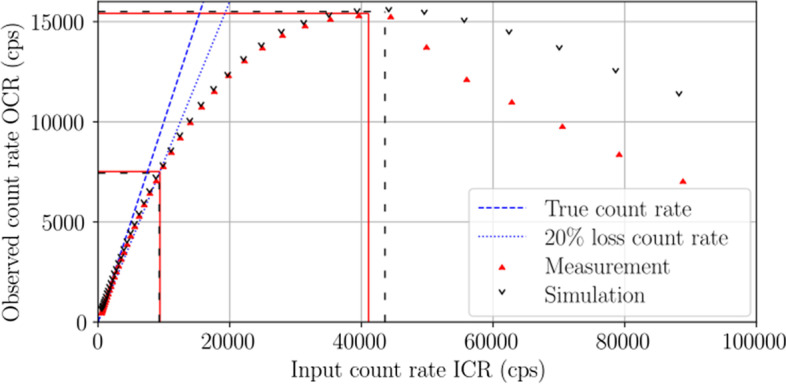
Intrinsic count rate performance in air. Measured results are shown with red solid carets and simulated results with black empty carets. Additional lines illustrate the maximum OCR and 20% loss OCR for measurement (solid lines) and simulation (dashed lines). The count rates are in agreement below the maximum OCR, while above the maximum, the measured OCR falls off the trend line as the photopeak shifted to lower energies

Figure 4 presents the $^{99\text {m}}$Tc count rate performance curve where the measured (simulated) maximum and 20% loss OCRs were 15,410 cps (15,500 cps) and 7,520 cps (7,440 cps), respectively. The measured data were corrected for uniformity and a background count rate of 11.6 cps to directly compare with the simulation for which no corrections were necessary. The measured and simulated results were comparable at input count rates below the maximum. However, the experimental detector did not behave like an idealized paralyzable system at relatively large count rates. Unexpected behaviour was observed through photopeak shifting in addition to pulse pile-up and dead time effects at count rates beyond the maximum—a count rate range unlikely to be encountered with typical *in vivo* usage of the Spark. The measured (simulated) dead time was found to be 23.9 $\mu$s (23.8 $\mu$s) using Eq. 4.

#### Intrinsic energy resolution

**Fig. 5:**
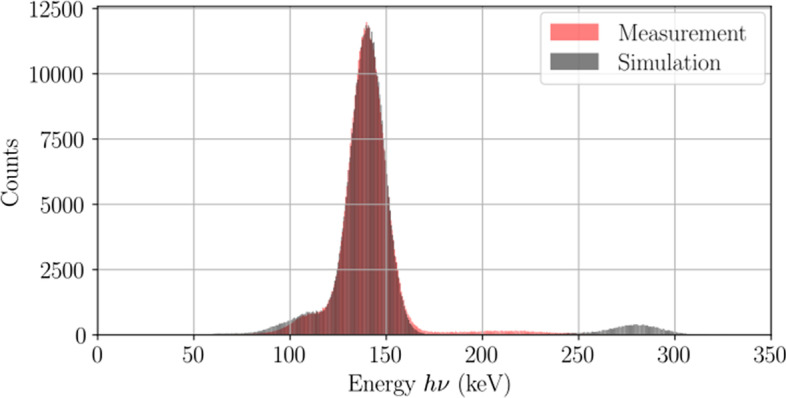
Measured and simulated $^{99\text {m}}$Tc energy spectra acquired at a count rate loss below 20%. The intrinsic resolution was 14.7% in both cases. Differences can be observed in the pile-up energy distribution due to partial scintillation light collection of the SiPM array, which was not modelled with GATE

Energy spectra are presented in Fig. 5 where the intrinsic energy resolution was 14.7% in measurement and simulation. Minute differences can be observed in the energy spectra at energies above the photopeak due to incomplete scintillation light collection during pile-up in the experimental system. Aside from the differences in pile-up energy distribution, a 3.1% difference was found in the number of pile-up events detected in an energy window extending above 150 keV.

### Tests of gamma camera detectors with collimators

#### System spatial resolution without scatter

**Fig. 6:**
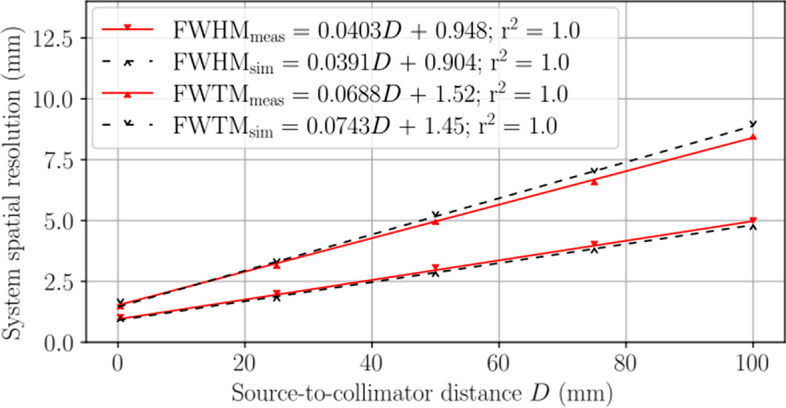
System spatial resolution without scatter presented in terms of FWHM and FWTM for the SPH collimator. Measured results are shown with solid red carets and solid lines of best fit, and simulated results are shown with empty black carets and dashed lines of best fit. Equations for lines of best fit are distinguished in the legend with abbreviated subscripts. The measured (simulated) system resolution without scatter at the center of rotation was 1.87 mm (1.80 mm)

The system spatial resolution without scatter is shown in Fig. 6. A linear least squares fit to the measured and simulated data calculated a coefficient of determination of $r^2=1.0$ and similar FWHM line equations. The FWHM equations predicted a measured (simulated) limiting system spatial resolution of 1.87 mm (1.80 mm) at the center of rotation ($D=23.0$ mm). Overall, the FWHM differences between measurement and simulation varied from 4.8% to 3.2% over source-to-collimator distances from 0 mm to 100 mm, respectively. A discrepancy can be observed in the FWTM best-fit lines.

#### System spatial resolution with scatter

**Fig. 7:**
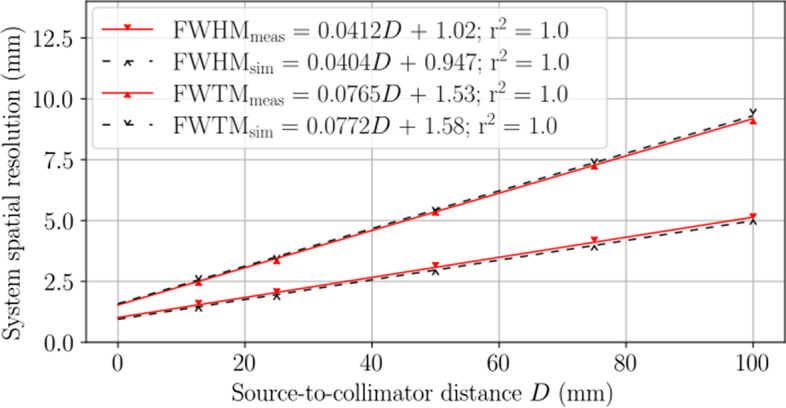
System spatial resolution with scatter in a mouse-sized NEMA triple line source scatter phantom presented in terms of FWHM and FWTM for the SPH collimator. Measured results are shown with solid red carets and solid lines of best fit, and simulated results are shown with empty black carets and dashed lines of best fit. Equations for lines of best fit are distinguished in the legend with abbreviated subscripts. The measured (simulated) system resolution with scatter at the center of rotation was 1.97 mm (1.88 mm)

The system spatial resolution with scatter in the mouse-sized NEMA triple line source scatter phantom is presented in Fig. 7. Linear least squares fits calculated a coefficient of determination of $r^2=1.0$ and comparable FWHM and FWTM fit equations between measurement and simulation. The FWHM equations predicted a measured (simulated) system spatial resolution with scatter of 1.98 mm (1.88 mm) at the center of rotation. Here, the FWHM differences between measurement and simulation varied from 7.1% to 2.9% over source-to-collimator distances from 0 mm to 100 mm, respectively. Interestingly, the FWTM best-fit lines have a higher degree of correspondence between measurement and simulation with scatter than without.

#### System planar sensitivity

**Fig. 8:**
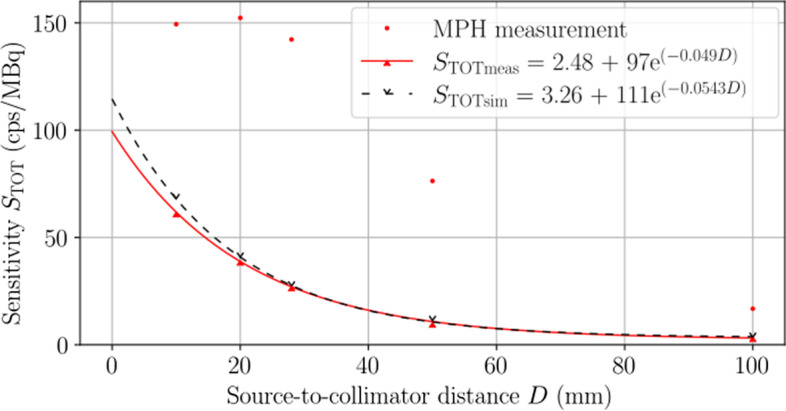
Planar sensitivity as a function of source-to-collimator distance. For the SPH collimator, measured results are shown with solid red carets and a solid line of best fit, and simulated results are shown with black empty carets and a dashed line of best fit. MPH collimator results are shown as red dots. Fit equations for the SPH collimator measurement and simulation are distinguished in the legend with abbreviated subscripts. The MPH collimator geometry is optimized for increased sensitivity in the tomographic FOV, whereas the SPH collimator sensitivity increases when approaching the pinhole

The total system planar sensitivity is presented in Fig. 8 for the SPH and MPH collimators. For the SPH collimator, the exponential fit calculated a measured (simulated) planar sensitivity of 33.8 cps/MBq (35.2 cps/MBq) at the center of rotation, reflecting a 4.0% difference. The difference increased to 14.2% at the face of the collimator, which could be partly due to limitations in modelling the collimator with a 0.85 mm pinhole. For the MPH collimator, sensitivity is optimized within the tomographic FOV due to the focusing nature of the pinholes. Therefore, the three largest values were fit with a quadratic function, and the planar sensitivity interpolated at the center of rotation was 150 cps/MBq.

### Tests specific to tomographic camera systems

#### SPECT reconstructed spatial resolution without scatter

Table 4 details the three-dimensional (3D) resolution results from all reconstructed point source images. Acquisitions with the SPH collimator calculated a measured (simulated) limiting 3D resolution of $1.37\pm 0.15$ mm ($1.30\pm 0.15$ mm). The MPH collimator yielded a 13% improvement in the limiting 3D resolution with a value of $1.19\pm 0.20$ mm and a submillimeter tangential resolution ($0.91\pm 0.05$ mm) due to the lateral focusing pinholes. Note that leaching of radioactivity into the capillary tube sealing clay was observed in measured tomographic images. A closer inspection of Table 4 confirms that the axial resolutions were overestimated in measurement when considering that all other FWHM were nearly identical between SPH collimator measurement and simulation.

**Table 4:** SPECT reconstructed spatial resolution without scatter

Reporting parameter	Measurement			Simulation	
Collimator	MPH	SPH	SPH	SPH	SPH
Reconstruction algorithm	MLEM	MLEM	FBP	MLEM	FBP
Central transaxial FWHM (*x*, *y*) (mm)	$1.29\pm 0.04$	$1.45\pm 0.01$	$2.26\pm 0.01$	$1.50\pm 0.01$	$2.26\pm 0.01$
Central axial FWHM (*z*) (mm)	$1.56\pm 0.01$	$1.58\pm 0.01$	$2.45\pm 0.01$	$1.31\pm 0.01$	$2.26\pm 0.01$
Peripheral radial FWHM (*x*) (mm)	$1.13\pm 0.08$	$1.25\pm 0.06$	$2.03\pm 0.10$	$1.30\pm 0.09$	$2.11\pm 0.25$
Peripheral tangential FWHM (*y*) (mm)	$0.91\pm 0.05$	$1.23\pm 0.11$	$1.94\pm 0.14$	$1.26\pm 0.13$	$1.93\pm 0.10$
Peripheral axial FWHM (*z*) (mm)	$1.24\pm 0.04$	$1.45\pm 0.16$	$2.70\pm 0.04$	$1.12\pm 0.06$	$2.48\pm 0.05$
Average 3D FWHM (mm)	$1.19\pm 0.20$	$1.37\pm 0.15$	$2.26\pm 0.30$	$1.30\pm 0.15$	$2.20\pm 0.23$

#### SPECT reconstructed spatial resolution with scatter

**Fig. 9:**
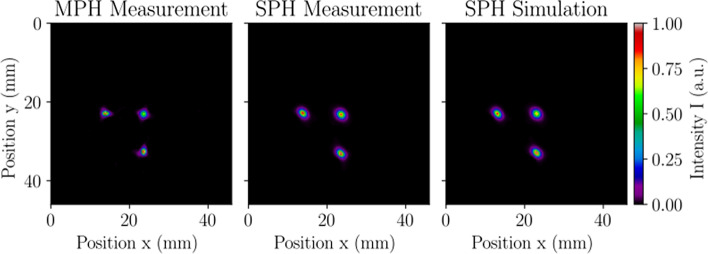
SPECT reconstructed spatial resolution with scatter evaluated with a mouse-sized NEMA triple line source scatter phantom in MPH collimator measurement (left), SPH collimator measurement (middle), and SPH collimator simulation (right). The images present the central 3.5 mm-thick transverse slice from the MLEM reconstruction used in calculating the radial, tangential, and central resolution. Images were normalized by the maximum displayed pixel value, resulting in intensity values with arbitrary units (a.u.). The MPH collimator offers superior tomographic resolution compared to the SPH collimator when scanning relatively small distributions of radioactivity

Figure 9 presents the central 3.5 mm-thick slice of the mouse-sized NEMA triple line source scatter phantom from the MLEM reconstructions, and Table 5 gives a breakdown of the in-plane resolution values from all reconstructed mouse phantom images. Acquisitions with the SPH collimator produced a measured (simulated) average in-plane resolution of $1.44\pm 0.07$ mm ($1.46\pm 0.07$ mm), and the MPH collimator yielded a 17% improvement with an average FWHM of $1.18\pm 0.15$ mm. Measurement and simulation were found to have an excellent agreement in tomographic resolution with differences below 2%. Although the MPH collimator is capable of higher resolution than the SPH collimator, the reduced standard deviation of the SPH resolution indicates that its in-plane resolution is more symmetric throughout the tomographic FOV.

**Table 5:** SPECT reconstructed spatial resolution with scatter

Reporting parameter	Measurement			Simulation	
Collimator	MPH	SPH	SPH	SPH	SPH
Reconstruction algorithm	MLEM	MLEM	FBP	MLEM	FBP
Central FWHM (mm)	$1.29\pm 0.05$	$1.52\pm 0.04$	$2.23\pm 0.07$	$1.54 \pm 0.05$	$2.21 \pm 0.08$
Radial FWHM (mm)	$1.27\pm 0.06$	$1.39\pm 0.06$	$2.34\pm 0.11$	$1.40\pm 0.04$	$2.30\pm 0.06$
Tangential FWHM (mm)	$0.99\pm 0.07$	$1.41\pm 0.03$	$2.03\pm 0.11$	$1.43 \pm 0.03$	$2.06 \pm 0.10$
Average in-plane FWHM (mm)	$1.18\pm 0.15$	$1.44\pm 0.07$	$2.20\pm 0.16$	$1.46 \pm 0.07$	$2.19 \pm 0.12$

#### SPECT volume sensitivity, uniformity, and variability

**Fig. 10:**
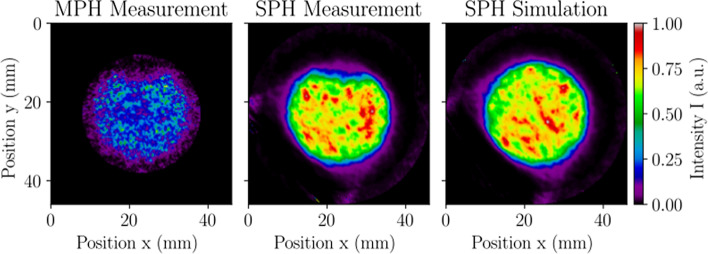
SPECT volume sensitivity, uniformity, and variability evaluated with a cylindrical phantom in MPH collimator measurement (left), SPH collimator measurement (middle), and SPH collimator simulation (right). The images present the central 0.25 mm-thick transverse slice from the MLEM reconstruction used in calculating volume uniformity and variability. Images were normalized by the maximum displayed pixel value, resulting in intensity values with arbitrary units (a.u.). A bubble can be seen in the measured data that was not modelled in the simulation. The SPH collimator offers a larger FOV with superior uniformity and noise characteristics compared to the MPH collimator when scanning relatively large distributions of radioactivity

Tomographic images of the cylindrical phantom reconstructed with the MLEM algorithm are presented in Fig. 10, and the corresponding volume sensitivity, uniformity, and variability results are given in Table 6. When comparing the measurement to simulation, the SPH volume sensitivity had the largest discrepancy observed across all NEMA tests, with a difference of 7.3%. This can be attributed to the slight overestimation in simulated sensitivity, an air bubble in the phantom during measurement that increased the source-to-collimator distance on average, and the exclusion of the animal bed from the simulation model. Although the SPH collimator has fewer pinholes than the MPH collimator and utilizes a smaller area of the UFOV, its increased tomographic FOV and total system response compensate for the relatively low sensitivity. Furthermore, tomographic images produced with the SPH collimator are considerably more uniform with less variability than those made with the MPH collimator.

**Table 6:** SPECT volume sensitivity, uniformity, and variability

Reporting parameter	Measurement		Simulation
Collimator	MPH	SPH	SPH
System volume sensitivity SVS (cps/(MBq/$\hbox {cm}^3$))	2200	329	354
Volume sensitivity per axial centimeter VSAC (cps/(MBq/$\hbox {cm}^2$))	1570	157	169
Total system response (cps/(MBq/$\hbox {cm}^3$))	2200	901	970
Uniformity (%)	96.5	48.2	38.2
Coefficient of variation CV (%)	38.7	11.6	10.2

## Discussion

The performance characteristics of a high-resolution SiPM-based preclinical SPECT system—the Cubresa Spark—have been evaluated for the first time according to the NEMA NU 1-2018 Standard for Performance Measurements of Gamma Cameras. The primary challenge in applying the NEMA NU 1 standard in a preclinical setting with a small-area detector was satisfying count-related specifications in the MWSR and SPECT reconstructed spatial resolution tests. Despite the relatively low count statistics associated with the SPH collimator and pencil beam apertures, practical count-starved acquisitions were obtained in favour of timely measurements because adherence to count criteria was inherently so time-consuming that it was considered unduly burdensome. One test that exceeded the count criteria was the intrinsic count rate performance in air which specifies that the final data point should be measured when the observed count rate drops below 4,000 cps because the dead time is only a fraction of a percent. Adherence to this specification would have yielded an overestimated 20% loss count rate of 10,000 cps due to a failure to extract the true input count rate from the linear response region of the detector.

Upon comparison with available reference values from Cubresa, the measured intrinsic spatial resolution of $0.851\pm 0.010$ mm was in excellent agreement with the reference value of 0.85 mm. To our knowledge, this is the highest reported intrinsic resolution of any $\gamma$-camera evaluated with the NEMA NU 1 standard to date. When comparing the system planar sensitivities, measured results of 33.8 cps/MBq for the SPH collimator and 150 cps/MBq for the MPH collimator were not in agreement with the respective reference values of 50 cps/MBq and 467 cps/MBq. The discrepancy is likely due to differences in source geometry for which a planar source was used in this study, while Cubresa likely used a point source. In general, pinhole collimator sensitivity is greatest along the pinhole axis and decreases when moving orthogonally off-axis. Therefore, the measured and simulated SPH collimator sensitivity represents the average sensitivity in a 35 mm-diameter plane located 23 mm from the face of the collimator. Furthermore, the sensitivity profiles shown in Fig. 8 underestimate the sensitivity for source positions near the face of the collimator due to the extent of radioactivity lying outside of the conic pinhole FOV. Regarding the MPH collimator and recalling that each row of pinholes focuses on a different VOI, accurate measurement of the MPH collimator sensitivity would require optimal placement of separate sources centered at the focal point of each VOI to ensure that all emissions occur inside the conic FOVs of all pinholes. This could be done with detailed knowledge of pinhole geometry.

When comparing internal results between measurement and simulation, the intrinsic performance parameters were very similar, and measured results were accurately simulated, which primarily validates the GATE detector head and digitizer settings. The parameters set in the digitizer differ from the corresponding observables, highlighting the importance of tuning the digitizer—a complex achievement with the Spark since it is not a conventional $\gamma$-camera. This process was made simpler and more accurate by applying NEMA’s methodology. Comparisons of the system and SPECT performance also showed excellent agreement between measurement and simulation, with the most considerable differences amounting to $\lesssim$ 7%. Altogether, these results confirm the accuracy of the Monte Carlo simulation results and satisfy the secondary objective of validating the GATE simulation model of the Spark for use in preclinical SPECT studies, such as validating STIR’s pinhole-SPECT image reconstruction capabilities [24, 28].

When tuning the GATE model for the system and tomographic simulations, a 0.85 mm-diameter pinhole was defined with a $90^{\circ }$ acceptance angle for the SPH collimator to better match the measured and simulated collimator-detector response functions in terms of resolution and sensitivity. This diameter was obtained following the interpolation methodology for calculating digitizer parameters as described in the Simulation description subsection. When simulating a 1.0 mm-diameter pinhole, the simulated system resolution without scatter was characterized as $\text {FWHM}=0.0424D + 1.12$ (mm), which agrees well with theoretical equations from Van Audenhaege et al. [38], but predicts a limiting resolution of 2.10 mm at the center of rotation that does not correspond with the measured result of 1.87 mm. This discrepancy can be attributed to a vendor-specific event positioning algorithm that improves the Spark’s resolution, which could not be accounted for using the digitizer. Similarly, the simulated 1.0 mm pinhole system planar sensitivity was characterized as $S_\text {TOT}=4.33 + 148\textrm{e}^{(-0.0563D)}$ (cps/MBq), which predicts a sensitivity of 44.9 cps/MBq at the center of rotation which is much greater than the measured result of 33.8 cps/MBq. This discrepancy could relate to the choice of CsI as the scintillation crystal material. This predefined material describes unactivated CsI and has the same physical characteristics as CsI(Na) and thallium-activated CsI (CsI(Tl)), but differs in terms of optical properties such as scintillation light yield, de-excitation time, refractive index, and peak emission wavelength [39, 40]. The introduction of optical properties into the simulation could allow for simulation of the entire SiPM readout logic to improve sensitivity and overall simulation accuracy. However, it would significantly increase computation time. Current efforts are ongoing to incorporate SiPM-specific software into GATE’s digitizer to reproduce signals from SiPMs [41, 42].

Several commercially available preclinical SPECT systems have been validated with GATE using ${}^\text {99m}$Tc and are compared in Table 7. Comparisons with pinhole collimators are made against 1.0 mm-diameter pinholes where data were available except for the NanoSPECT/CT$^{\textrm{PLUS}}$ which uses 1.5 mm pinholes. System and SPECT parameters are cited at the radius of rotation. These tabulated studies not only demonstrate the flexibility and reliability of GATE for accurately modelling various detector designs but also illustrate the potential of SiPMs in molecular imaging. Comparisons of $\gamma$-camera performance for different imaging systems are best performed according to the NEMA NU 1 standard as it provides a uniform and consistent method for measuring and reporting performance parameters for various camera designs. Unfortunately, most tabulated systems were not evaluated with NEMA standards, perhaps due to the absence of a dedicated preclinical SPECT standard from NEMA, a shortage of required resources, or a restriction from essential scanner data. Therefore, direct comparisons are limited due to inconsistent reporting parameters from different researchers and organizations. Nonetheless, this study has demonstrated competitive performance characteristics of the novel SiPM-based SPECT system, including the highest intrinsic spatial resolution of the tabulated $\gamma$-cameras, the smallest form factor, good energy resolution, and comparable sensitivity and tomographic resolution to the top-performing preclinical systems.

**Table 7:** Performance comparisons of commercial preclinical SPECT cameras validated with GATE using ${}^{99\text {m}}$Tc

Reporting parameter	Spark	X-SPECT	Inveon	HiReSPECT	NanoSPECT
Detection method	SiPM	CZT	PSPMT	PSPMT	PMT
Scintillation crystal^a^	CsI(Na)	N/A	NaI(Tl)	CsI(Na)	NaI(Tl)
Collimator^b^	SPH/MPH	SPH/MPH	SPH/MPH	PH	SPH/MPH
Radius of rotation (mm)	28	25	25	25	45
Magnification factor	$\sim 1\times$	$\sim 4\times$	$\sim 4\times$	$1\times$	$\sim 3.5\times$
Energy window width	30%	20%	20%	N/A	20%
Aperture size (mm)	1.0	1.0	1.0	1.2	1.5
Intrinsic spatial resolution (mm)	0.85	1.5	N/A	N/A	3.2
Intrinsic energy resolution (%)	14.7	5	12.4	19.15	8.7
System resolution (mm)	SPH: 1.87	SPH: 1.02	N/A	2.79	N/A
Sensitivity (cps/MBq)	SPH: 34	MPH: 155	SPH: 38	36–42	SPH: 42
	MPH: 150		MPH: 286		MPH: 191
SPECT resolution (mm)	SPH: 1.37	MPH: 0.58	SPH: 1.25	1.7	SPH: 1.27
	MPH: 1.19				MPH: 1.24
References	N/A	[14, 43, 44]	[15, 45–47]	[16, 48, 49]	[17, 50]

^a^ CsI(Na): sodium-activated cesium iodide, NaI(Tl): thallium-activated sodium iodide

^b^ SPH: single-pinhole, MPH: multi-pinhole, PH: parallel-hole

N/A: not applicable or not available

## Conclusion

The performance of a novel, preclinical SiPM-based SPECT scanner has been characterized according to the NEMA NU 1-2018 Standard for Performance Measurements of Gamma Cameras. Measured and simulated NEMA tests were highly comparable, where the most considerable differences were below 7%, and overall differences were a few percent. This confirms simulation accuracy and satisfies the secondary objective of validating the GATE Monte Carlo model. Of the collimators initially provided with the Spark, the multi-pinhole collimator investigated in this study offers increased spatial resolution and sensitivity for organ-specific imaging of small animals, and the single-pinhole collimator enables high-resolution whole-body imaging of small animals. This work demonstrates that a SiPM detector mitigates the need for highly magnifying collimators while preserving detailed information in projection images.

## Data Availability

The datasets used and/or analyzed during the current study are available from the corresponding author on reasonable request.

## Abbreviations

3D: Three-dimensional
$$^{57}$$Co: Cobalt-57
$$^{67}$$Ga: Gallium-67
$$^{99\text {m}}$$Tc: Technetium-99m
CFOV: Central field of view
CsI(Na): Sodium-activated cesium iodide
CsI(Tl): Thallium-activated cesium iodide
CT: Computed tomography
CV: Coefficient of variation
CZT: Cadmium zinc telluride
EGS: Electron Gamma Shower
FBP: Filtered back projection
FOV: Field of view
FWHM: Full width at half maximum
FWTM: Full width at tenth maximum
GATE: Geant4 Application for Tomographic Emission
Geant4: Geometry and Tracking
ICR: Input count rate
LSF: Line spread function
Meas: Measurement
MLEM: Maximum likelihood expectation maximization
MCNP: Monte Carlo N-Particle
MPH: Multi-pinhole
MWSR: Multiple window spatial registration
NaI(Tl): Thallium-activated sodium iodide
NEMA: National Electrical Manufacturers Association
OCR: Observed count rate
PET: Positron emission tomography
PH: Parallel-hole
PMT: Photomultiplier tube
PSF: Point spread function
PSPMT: Position sensitive photomultiplier tube
ROI: Region of interest
Sim: Simulation
ROOT: Rapid Objected-Oriented Technology
SiPM: Silicon-photomultiplier
SPECT: Single-photon emission computed tomography
SPH: Single-pinhole
STIR: Software for Tomographic Image Reconstruction
STL: Standard tessellation language
SVS: System volume sensitivity
UFOV: Useful field of view
VOI: Volume of interest
VSAC: Volume sensitivity per axial centimeter
